# Comparing the angiogenic potency of naïve marrow stromal cells and Notch-transfected marrow stromal cells

**DOI:** 10.1186/1479-5876-11-81

**Published:** 2013-03-27

**Authors:** Mo Dao, Ciara C Tate, Michael McGrogan, Casey C Case

**Affiliations:** 1 SanBio Inc., 231 South Whisman Road, Mountain View, CA, USA

**Keywords:** Marrow stromal cells, SB623 cells, Angiogenesis, Stroke, Aortic ring assay, HUVECs

## Abstract

**Background:**

Angiogenesis is a critical part of the endogenous repair process in brain injury and disease, and requires at least two sequential steps. First, angiogenic sprouting of endothelial cells occurs, which entails the initial proliferation of endothelial cells and remodeling of the surrounding extracellular matrix. Second, vessel stabilization is necessary to prevent vascular regression, which relies on vascular smooth muscle recruitment to surround the young vessels. Marrow stromal cells (MSCs) have been shown to promote revascularization after hindlimb ischemia, cardiac ischemia, and stroke. SB623 cells are derived from marrow stromal cells by transfection with a *Notch1 intracellular domain* (NICD)-expressing plasmid and are known to elicit functional improvement in experimental stroke. These cells are currently used in human clinical testing for treatment of chronic stroke. In the current study, the angiogenic property of SB623 cells was investigated using cell-based assays.

**Methods:**

Angiogenic paracrine factors secreted by SB623 cells and the parental MSCs were identified using the Qantibody Human Angiogenesis Array. To measure the angiogenic activity of conditioned medium from SB623 cells and MSCs, endothelial tube formation in the human umbilical vein endothelial cell (HUVEC) assay and endothelial cell sprouting and branching in the rodent aortic ring assay were quantified. To validate the angiogenic contribution of VEGF in conditioned medium, endothelial cells and aortic rings were treated with SU5416, which inhibits VEGFR2 at low dose.

**Results:**

Conditioned medium from SB623 cells promoted survival and proliferation of endothelial cells under serum-deprived conditions and supports HUVEC vascular tube formation. In a rodent aortic ring assay, there was enhanced endothelial sprouting and branching in response to SB623-derived conditioned medium. SU5416 treatment partially reversed the effect of conditioned medium on endothelial cell survival and proliferation while completely abrogate HUVEC tube formation and endothelial cell sprouting and branching in aortic ring assays.

**Conclusions:**

These data indicate that SB623 cell-secreted angiogenic factors promoted several aspects of angiogenesis, which likely contribute to promoting recovery in the injured brain.

## Background

Angiogenesis is the formation of new blood vessels from existing vasculature. In the adult, angiogenesis occurs after brain injury as part of the repair and regeneration process. This process acutely functions to both aid clearance of dead tissue and restore nutrients to surviving tissue. In the sub-acute and chronic phases, angiogenesis accompanies neurogenesis during regeneration [1,2]. The angiogenesis process includes basement membrane disruption, endothelial cell migration and proliferation, three-dimensional tube formation, maturation, and stabilization by vascular smooth muscle cells. Each step is regulated by multiple cytokines and extracellular matrix molecules [3,4]. It is well-established that factors secreted by mesenchymal stromal cells (MSCs) from different sources regulate angiogenesis. For example, endometrial MSCs promote angiogenesis after critical hindlimb ischemia through secretion of growth factors and matrix metalloproteinases [5]. Bone marrow derived MSC-conditioned medium enhances endothelial cell and smooth muscle cell proliferation and migration [6,7] and promotes angiogenesis after hindlimb ischemia [8]. In myocardial ischemic models, MSCs injected intravenously or intracardially increase neovascularization and improve heart function [9,10]. Transplantation of MSCs also increases angiogenesis following experimental ischemic stroke [11-13] and traumatic brain injury [14].

SB623 cells are derived from marrow stromal cells transfected with a plasmid expressing the human *Notch1 intracellular domain (NICD1*) [15]. SB623 cells express all of the standard mesenchymal stromal cell markers such as CD90, CD44, and CD105 and the expression of NICD1 is transient [16]. Compared to MSCs, SB623 cells secrete higher levels of various paracrine factors such as IL-6, IL-8, and MCP-1 [17]. SB623 cells have been shown to reduce the lesion volume and promote functional recovery when delivered to rodent brains following experimental focal ischemia [18]. Currently in an FDA-approved Phase I/IIa clinical trial for chronic stroke, SB623 cells are delivered stereotactically to the perinfarct region (clinicaltrials.gov/ct2/show/NCT01287936). While SB623 cell transplantation clearly aids the injured brain, the exact mechanisms are yet to be elucidated. In the current study, we compared the angiogenic potency of SB623 -conditioned medium with the parental MSC-conditioned medium using various *in vitro* models. In addition, we begin to determine which secreted soluble cytokines may be involved.

## Methods

### Materials

All reagents are from Life Technologies (Carlsbad, CA) unless indicated otherwise. Human umbilical vein cells (HUVECs) and Sprague–Dawley rats were purchased from ATCC (Manassas, VA) and Charles Rivers Laboratories (Wilmington, MA), respectively.

### Culture of MSCs and SB623 cells and preparation of conditioned medium

Two human cell types were examined in this study; mesenchymal stromal cells (MSCs) and MSC-derived SB623 cells. Bone marrow aspirates from healthy human adults were obtained from Lonza (Walkersville, MD), rinsed, and plated in tissue culture flasks. Culture medium for the generation and maintenance of donor cells was minimum essential alpha medium (α - MEM, Mediatech, Herndon, VA) supplemented with 2mM Glutamine, 10% fetal bovine serum (Hyclone, Logan, UT) and 1% penicillin/streptomycin (referred to throughout the text as “growth medium”). Non-adherent cells were discarded, and the remaining cells were passed two times using trypsin (0.25% + 2 mM EDTA). MSCs were then either frozen for later use or plated for SB623 cell preparation. For SB623 preparation, MSCs were transfected with the pCI plasmid expressing the human *Notch1 intracellular domain (NICD*; *Notch1* truncated at the transmembrane domain) and the neomycin-resistance gene using Fugene6 (Promega, Madison, WI) according to the manufacturer’s protocol. The next day, the medium was replaced with growth medium containing 100 μg/ml G418 and selection continued for 7 days. Selection medium was then replaced with growth medium. After removal of G418 and recovery, cells were passed two additional times. SB623 cells were harvested using trypsin-EDTA and cryopreserved for later use. Both MSCs and SB623 cells were routinely characterized by flow cytometry analysis and were found to be positive (>95%) for CD29, CD90, and CD105, and negative (<5%) for CD31, CD34, CD45, indicating their mesenchymal nature. For experiments, frozen MSCs and SB623 cells from the same human donor were thawed, re-plated and allowed to recover for approximately one week. To obtain conditioned medium, MSCs or SB623 cells were cultured in growth medium to ~90% confluence (~15,000 cells/cm^2^). Following rinsing in phosphate-buffered saline (PBS), the medium was replaced with Opti-MEM® medium (~150,000 cells/ml), and the conditioned medium was collected 72 hours later and stored at -80°C. At the time of collection, the number of cells was quantified (mean = 1.0 ± 0.1 million cells per flask, with no significant differences between MSCs and SB623 cells). Frozen conditioned medium samples were slowly warmed to 37°C on the day of experimentation.

### Angiogenic cytokine array of MSC- and SB623-conditioned medium

To identify angiogenic trophic factors secreted by MSCs and SB623 cells, the protein levels of specific factors in donor cell-conditioned medium were measured. The Quantibody® Human Angiogenesis Array 1 (RayBiotech, Norcross, GA) was used to determine the concentrations of the following 10 cytokines in MSC or SB623 cell-conditioned media: angiogenin, angiopoietin-2, epidermal growth factor (EGF), fibroblast growth factor–2 (FGF-2/ bFGF), heparin binding-epidermal growth factor like growth factor (HB-EGF), hepatocyte growth factor (HGF), leptin, platelet derived growth factor-BB (PDGF-BB), placental growth factor (PIGF), and vascular endothelial growth factor (VEGF). To account for slight differences in actual cell numbers, the data were normalized to the cell number determined upon collecting conditioned medium, and are thus expressed as protein concentration per 1 million cells. For this assay, 4 different human donor pairs were tested.

### Cell proliferation and survival of HUVECs

Two different lots of human umbilical vein endothelial cells (HUVECs) were purchased from ATCC. Experiments comparing SB623-CM and MSC-CM were performed with one lot of HUVECs; experiments assessing the effect of SU5416 on SB623-CM angiogenic activity were performed with a second lot of HUVECs. Briefly, HUVECs were plated in endothelial basal medium-2/10%FBS plus endothelial cell growth supplement (EBM-2/10%FBS plus ECGS) at 750,000 cells per 0.1% gelatin-coated T-75 flask for 24 hours. To prepare for the serum-growth factor withdrawal study, HUVEC monolayers were rinsed twice with warm PBS and incubated with fresh 12 ml EBM-2 medium for 16–24 hours at 37°C, 5% CO_2_. Serum-growth factor withdrawal was initiated by re-feeding each flask with 6 ml of EBM-2 medium plus 6 ml OptiMEM (control) or 6 ml of EBM-2 medium supplemented with 6 ml of either MSC- or SB623 cell-derived conditioned OptiMEM. After 7 days, non-adherent and adherent cells were collected, centrifuged at 1400 rpm for 5 minutes, and divided into three fractions for the subsequent three staining analyses.

To quantify cell death, cells were stained with 5 μg/ml of propidium iodide (PI) for 30 min at room temperature and acquisition/analysis was done on FL-2 logarithmic channel using BD FACS Calibur CellQuest program. Staining for Bcl-2 (anti-apoptotic molecule) and Ki67 (proliferation marker; BD Bioscience) was also performed. Cells were fixed in 4% paraformaldehyde and permeabilized with 0.1% Triton-X for one hour; after staining with either fluorescein-conjugated antibody against Bcl-2 or Ki67 for one hour on ice, samples were washed, acquired, and analyzed on FL-1 channel on BD FACS Calibur. For these assays, 3 different human donor pairs were tested with 3 wells per group.

For experiments with VEGFR inhibitor, HUVECs were pre-treated with 50 nM SU5416 (EMD Millipore, Billerica, MA) in EBM-2 medium for 30 minutes. Medium was then changed to 50 nM SU5416-supplemented medium containing 6 ml of EBM-2 medium plus 6 ml of SB623 cell derived-conditioned medium. Analyses for cell proliferation and cell death were performed after 5 days in culture. For these assays, SB623 cells from 3 different human donors were tested with 3 wells per group.

### HUVEC tube formation assay

HUVECs were cultured in α-MEM/0.5%FBS/2 mM glutamine/pen-strep for 24 hours and stained with 1 μM calcein dye (Life Technologies, Carlsbad, CA) prior to use in a vascular tube formation assay. In each well of a 96-well flat bottom plate, 50 μl of reduced growth factor (RGF)-Cultrex basement membrane extract (Trevigen, Gaithersburg, MD) were incubated at 37°C for 45 minutes. HUVECs were harvested using 0.25% trypsin-EDTA, rinsed, and re-suspended in α-MEM/2 mM glutamine/pen-strep at 1×10^5^ cells/ml. 75 μl of HUVECs plus 75 μl of either MSC- or SB623-conditioned medium (from 3 different human donor pairs) was added to each well. The negative control was HUVECs plus OptiMEM medium only. Fluorescent (for calcein-stained conditions) or phase contrast photographs were taken at 16 hours, for subsequent quantification of tube formation by an experimenter blinded to the groups. A continuous, unbroken ring of cells was considered a complete vascular tube. Where indicated, 50 nM SU5416 treatment was done 30 minutes prior to changing to 50 nM SU5416-supplemented SB623 cell derived-conditioned medium (from 3 different human donors), 50 nM SU5416-supplemented OptiMEM with 10 ng/ml recombinant human VEGF (positive control), or 50 nM SU5416-supplemented OptiMEM (negative control). For this assay, 3–4 wells/ group were used and the total number of tubes per well was quantified and averaged.

### Aortic ring assay

All procedures conformed to guidelines set forth in the NIH Guide for the Care and Use of Laboratory Animals. Adult Sprague–Dawley rats were euthanized prior to dissection. After opening the chest and clamping off the two ends, the aorta was removed and placed in ice-cold α-MEM/pen-strep medium prior to removal of the adipose external lining. Adipose-free aorta was rinsed twice with ice-cold EBM-2/pen-strep medium before sectioning into 1-mm-thick rings. The aortic rings were then transferred to plates containing α-MEM/2 mM glutamine/pen-strep medium and incubated at 37°C, 5% CO_2_ for 6 days to minimize the endogenous rat angiogenic factors; fresh medium was replaced on day 3. On day 6, the medium was replaced with α-MEM/pen-strep medium and the culture was continued for an additional 24-hour period. On day 0 of the aortic ring assay, 50 μl of RGF- basement gel was deposited per well of a 24-well plate. Each individual aortic ring was carefully placed in the middle of each well and over-layed with an additional 25 μl of RGF-Cultrex basement membrane extract. After the gel had solidified at 37°C, 5% CO_2_ for 30 minutes, 500 μl of α-MEM/2 mM glutamine/pen-strep was added to each well and incubated for an additional 30 minutes. To assess the angiogenic activity from MSC- and SB623-derived trophic factors, 500 μl of conditioned medium was added. As a negative control, 500 μl of unconditioned OptiMEM medium was used. Phase contrast photos were taken at 7 or 10 days and both vessel outgrowth and branching was quantified by an experimenter blinded to the experimental group. For this assay, 7 different human donor pairs were tested. To see the effect of VEGFR inhibitor on SB623-conditioned medium aniogenic activity, aortic rings were pre-treated with 50 nM SU5416 at 5% CO_2_/37degrees for 30 minutes; subsequently, medium was replaced with 50 nM SU5416-supplemented SB623-conditioned medium (from 1 representative donor). Each round of the assay included 3–4 wells per group (MSC-CM, SB623 CM, unconditioned medium, or SB623 CM + SU5416) that were quantified and averaged.

### Statistics

For each experiment (which included 3–4 wells/group), a mean value was obtained for: (1) the treatment condition for each cell type (either MSCs or SB623 cells; one value per human donor tested) and (2) the untreated group (one value for each round of testing). For statistical comparison (SigmaStat, StyStat Software, San Jose, CA) each of these values were used and comparisons were made using one way ANOVA between the following groups (1) Control (unconditioned medium; n = 3), (2) MSC-conditioned medium (n = 3-7); and (3) SB623 cell-conditioned medium (n = 3-7). Additional pair-wise comparisons were made using Tukey’s test. An alpha value of 0.05 was used to assess if the means were significantly different.

## Results

### MSCs and SB623 cells secrete multiple pro-angiogenic factors

**Figure 1:**
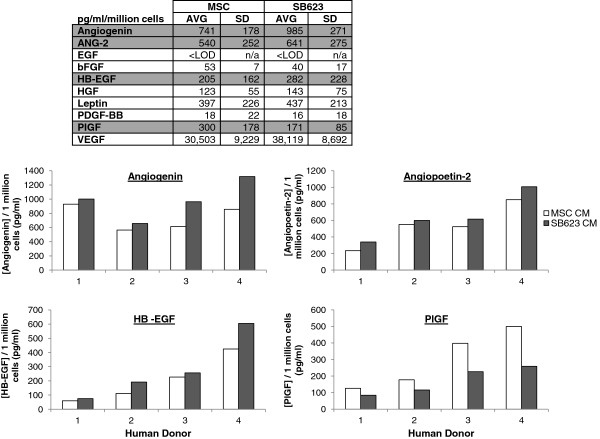
**MSCs and SB623 cells secrete pro-angiogenic factors. **The table shows average concentration of angiogenic cytokines found in OptiMEM conditioned for 72 hours by either MSCs or SB623 cells from 4 matched human donor lots. Note the relatively high expression of VEGF compared to other cytokines present. Grey highlights indicate factors which are consistently different between MSCs and SB623 cells. These 4 cytokines are plotted by donor showing that, though there is a lot of donor variability, there are consistent trends across multiple donors.

Previously, Tate et al. demonstrated that VEGF is among the trophic factors secreted in abundance by MSCs and SB623 cells [17]. In the current study, using an angiogenic cytokine array to compare SB623 cell- and its corresponding parental MSC-conditioned medium (CM) from four different human donors, we assessed the secretion of 10 angiogenic cytokines (expressed as protein concentration per million cells). Nine of the 10 angiogenic factors tested were detected in MSC- and SB623 cell-conditioned media (Figure 1). Due to high variability across donors, there were no significant differences in protein levels between SB623 CM and parental MSC-CM. However, angiogenin, angiopoetin-2, and HB-EGF were consistently found in higher levels in SB623-CM compared to their respective parent MSC-CM while PlGF was consistently lower for all four donors tested. Both MSCs and SB623 cells secrete relatively high levels of the prototypic angiogenic protein, VEGF, compared to the other proteins examined.

### MSC- and SB623-derived factors support HUVEC survival and proliferation

**Figure 2:**
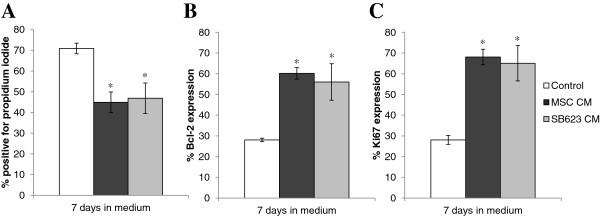
**SB623 cell- and MSC-conditioned medium supports HUVEC survival and proliferation.** Following “starvation”, HUVECs were cultured for 7 days with OptiMEM conditioned by either MSCs or SB623 cells or unconditioned OptiMEM (“Control”). **A**) Compared to unconditioned medium, conditioned medium (CM) led to a significant decrease in dead/dying propidium iodide-positive HUVECs. **B**) There was a significant increase in expression of Bcl-2, indicating that factors secreted by MSCs or SB623 cells are anti-apoptotic for HUVECs. **C**) Compared to unconditioned medium, CM led to a significant increase in Ki67-positive proliferating HUVECs. Mean ± SD; *p < 0.05 compared to OptiMEM only Control group.

An occlusion to the middle cerebral artery leads to a reduction in nutrients important not only to neural cells but also vascular cells. To determine if soluble factors from SB623 cells and MSCs have protective effect on nutrient-deprived endothelial cells, we cultured HUVECs in medium deprived of serum and growth factors for 24 hours prior to exposure to SB623- or MSC- conditioned medium (CM), or serum-reduced OptiMEM (negative control). After 5–7 days, cell death was assessed by propidium iodide staining and analyzed by flow cytometry. In the control condition where nutrient-deprived HUVECs were cultured in OptiMEM medium for 7 days, more than 70% of cells were positive for propidium iodide staining. Addition of either SB623- or MSC-conditioned medium significantly reduced the percentage of propidium iodide positive cells (p < 0.05; Figure 2A). Consistent with these results, we observed a significantly higher protein expression of Bcl-2, a pro-survival regulator, in HUVEC cells rescued by either SB623- or MSC-conditioned medium, compared to the control (p < 0.05; Figure 2B). By intracellular staining with a fluorochrome-conjugated antibody against Ki67, an indicator of cell division, followed by flow cytometry acquisition/analysis, we demonstrate that Ki67 expression was significantly increased when comparing the control cultures versus the SB623- or MSC-conditioned medium-rescue cultures (p < 0.05; Figure 2C). There were no significant differences in cell survival, Bcl-2, or Ki67 expression between HUVEC cultures rescued with SB623-CM versus MSC-CM. Collectively, the results demonstrate that SB623-conditioned medium is as effective as the parental MSC-conditioned medium in supporting the survival and proliferation of HUVECs.

### MSC- and SB623-derived factors enhance vascular tube formation

**Figure 3:**
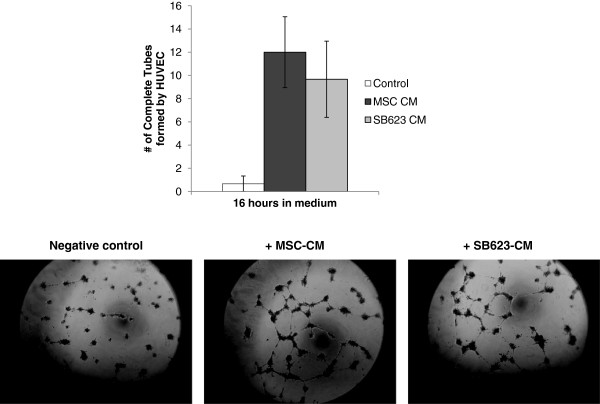
**SB623 cell and MSC-conditioned medium enhances HUVEC tube formation. **HUVECs were cultured with OptiMEM conditioned by either MSCs or SB623 cells or unconditioned OptiMEM (“Control”). Compared to unconditioned medium, there were more complete vascular tubes formed within 16 hours in the presence of conditioned medium (CM). The average number of completely-enclosed rings formed by the HUVECs (labeled “complete tubes”) in the entire well (96-well plate; 3–4 wells/group) is plotted. Mean ± SEM.

To re-establish the cerebral blood flow, the surviving and proliferating endothelial cells must receive external signals prompting their migration and invasion to form a complex vasculature. Using the well-established 3D-HUVEC vascular tube formation assay, we compared the potency of SB623 cell- versus MSC-conditioned medium in promoting complete vascular tubes after 16 hours in commercially-available, reduced-growth factor basement matrix gel. As shown in Figure 3, the addition of SB623-CM or MSC-CM increased the number of complete HUVEC tubes compared to the negative control. Similar to the above survival and proliferation results, there were no significant differences in the potency of SB623-CM versus MSC-CM in this process.

### MSC- and SB623-derived factors enhance angiogenesis in a rat aortic organotypic model

**Figure 4:**
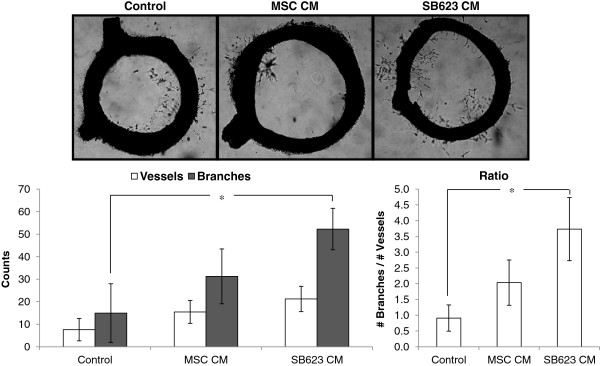
**Branching of new vessels is enhanced by MSC- and SB623 cell-secreted factors.** Following suppression of endogenous outgrowth, rat aortic rings were plated in gels with either OptiMEM (“Control”) or OptiMEM conditioned by MSCs or SB623 cells. After 7–10 days, there were more vessels with conditioned medium and significantly more branching points in SB623 CM versus OptiMEM only (*p < 0.05). Representative images of aortic rings after 7 days in culture with OptiMEM only, or MSC-conditoned medium, or SB623-conditioned medium. Mean ± SEM.

Under physiological conditions, endothelial cell proliferation and sprouting are activated by a spectrum of signals emanating from various cell types, including the monocyte/macrophages and the vascular smooth muscle cells. Therefore, to test the angiogenic potency of SB623 cell- and MSC-conditioned medium in a more physiologically relevant model than the HUVEC model, aortic rings from adult rats were generated. To minimize the production and carry-over of endogenous rat angiogenic factors induced in response to the slicing injury, the aortic rings were cultured in serum-free medium for a week prior to transfer onto a commercially available reduced-growth factor basement matrix for testing. After 7–10 days in the matrix, we noted a low number of sprouts and branches from the controls which were not exposed to CM of either SB623 cells or MSCs. In contrast, there was an increase in both the number of newly sprouted vessels and branching points from the aortic rings exposed to SB623 cell- and MSC-conditioned medium (Figure 4). Interestingly, there was a significantly higher amount of branching in cultures with SB623 cell-CM (but not with MSC-CM) compared to control conditions. These results demonstrate that, in a physiologically relevant 3D model, soluble factors from SB623 cells may be more potent in angiogenesis than soluble factors from the parental MSCs.

### Blocking VEGF signaling reduces but does not abrogate the angiogenic activity of SB623-CM

**Figure 5:**
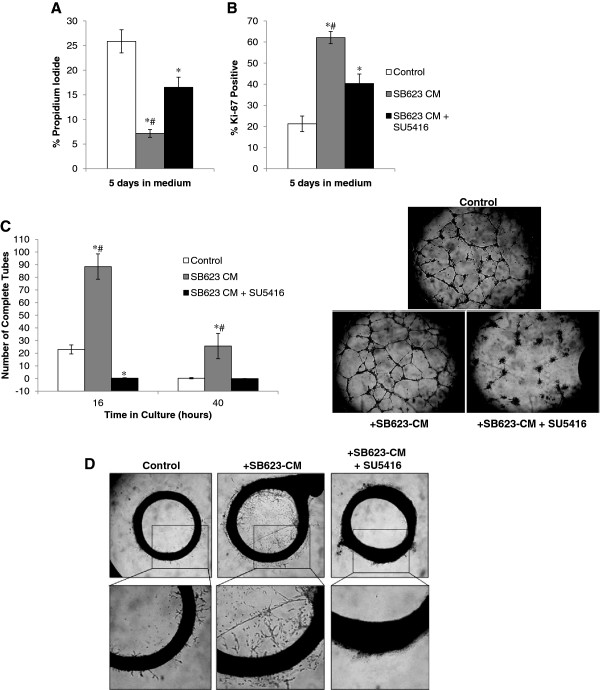
**Treatment with 50nM SU5416 reduces angiogenic potency of SB623 cell -conditioned medium. **Following “starvation”, HUVECs were treated with SB623-CM with or without SU5416 for 30 minutes prior to culture to assess the role of VEGF in different steps of angiogenesis. The negative controls were cultured directly in OptiMEM. **A **and **B**) Cell death and cell proliferation were assessed after 7 days in culture by staininig for propidium iodide and Ki67, respectively. SB623 cell-CM significantly reduced cell death and increased proliferation (*p < 0.05 versus OptiMEM only control). However, this was partially reversed in the presence of SU5416 (#p < 0.05 SB623 CM versus SB623 CM + SU5416). **C**) HUVEC vascular tube formation was quantified at 16- and 40 hours after plating in RGF-basement gel. A representative phase contrast picture of vascular tube formation at 16 hours is shown here, revealing the reduction in complete tubes formed in the presence of SU5416 (p < 0.05 versus OptiMEM only control; #p < 0.05 versus SB623 CM + SU5416). **D**) Aortic rings were likewise pre-treated with or without SU5416 for 30 minutes prior to embedding in RGF gels with SB623-CM. The control aortic ring well was not pre-treated with SU5416 and was maintained in RGF-basement gel in OptiMEM. It is clear that in the presence of SU5416 there is no aortic sprouting. Plots show Mean ± SD.

From the angiogenic cytokine array results, we demonstrate the presence of multiple angiogenic factors in addition to VEGF. To tease out the contribution of VEGF, we blocked VEGFR2 downstream signaling using SU5416 in the HUVEC and aortic ring assays to measure the angiogenic potency of non-VEGF cytokines present in SB623-CM. In the HUVEC model, a dose of 50 nM SU5416 has previously been shown to reduce VEGFR2-mediated endothelial cell proliferation with little impact on other receptor tyrosine kinases [19]. Here, we demonstrate that, pre-treatment with 50 nM SU5416 reduced, but did not abrogate, the pro-survival and proliferative activity of SB623 cell-CM on HUVECs (Figure 5A and B). In contrast, this dose was sufficient to reverse the ability of SB623 cell-CM to induce of HUVEC tube formation, highlighting the strong dependence of HUVEC cell migration and invasion on VEGFR2 activation (Figure 5C). Similarly, when applied to serum-starved aortic rings prior to addition of SB623 cell-CM, this dose completely prevented sprouting, again highlighting the VEGFR2-dependent pathway in this process (Figure 5D). These results underscore the complexity of angiogenic events with some steps being more dependent on VEGFR2 signaling than others.

## Discussion

Transplantation of SB623 cells, a derivative of marrow stromal cells, into animal models of stroke elicited functional recovery [18]. As there is strong correlation between angiogenesis and functional improvement in stroke patients [20], we hypothesized that the secretome of SB623 cells could include angiogenic factors. Using an angiogenic factor antibody array, we identified a panel of pro-angiogenic factors differentially secreted by SB623 cells and the corresponding parental MSCs. For instance, angiogenin, angiopoietin-2, HB-EGF, and VEGF levels were consistently detected at higher levels in SB623-conditioned medium than the parental MSC-conditioned medium, with VEGF being present at highest levels (>30 ng per million of SB623 cells) compared to the other three factors (< 1 ng per million of SB623 cells). Angiogenin has been reported to enhance endothelial cell invasiveness during migration by stimulating the proteolytic activities in cultured endothelial cells [21]. The presence of angiogenin in endothelial cells was suggested to be important for VEGF-mediated cell proliferation [22]. Angiopoietin-2 has been commonly described as an anti-angiogenic factor, destabilizing the vasculature and promoting endothelial cell death. However, in the presence of VEGF, angiopoietin-2 adopted the pro-angiogenic function, promoting the sprouting of new blood vessels as well as the increase in the capillary diameter [23]. Unlike angiogenin and angiopoietin-2, HB-EGF was shown to act on smooth muscle cells, stimulating their production of VEGF which in turn promotes endothelial cell proliferation [24]. Identification of these factors along with others such as PlGF, HGF, and leptin listed in Figure 5 supports the hypothesis that SB623 cells promote angiogenesis after stroke.

To validate the angiogenic activity present in SB623 secretome, we initiated various functional assays. First, to mirror the nutrient stress condition after stroke, we used a serum and growth factor-deprived model of HUVECs and measured the protective effect of SB623 on endothelial cells. By staining for propidium iodide (to measure cell death), Bcl-2 (an anti-apoptotic molecule), and Ki67 (an antigen present in non-quiescent cells), we demonstrated that conditioned medium from SB623 cells effectively promotes the survival and proliferation of nutrient-starved HUVECs; this effect was comparable to conditioned medium from MSCs. From the cytokine array results, VEGF is the angiogenic factor secreted at highest levels by MSC and SB623 cells. We therefore pre-treated HUVECs with 50 nM SU5416, a concentration previously reported to reduce VEGF mediated cell survival and proliferation without affecting the activity of other angiogenic factors such as bFGF or PDGF ([19]. The results demonstrated a 50% reduction in both cell survival and proliferation in SU5416 treated HUVECs cultured with conditioned medium from MSC or SB623 cells. The fact that we did not observe complete inhibition of cell survival and proliferation raises two possibilities. First, bFGF has been shown to be a potent mitogen and survival factor for endothelial cells [25]. Hence, the low levels of bFGF present in SB623-conditioned medium along with other angiogenic factors whose action did not depend on VEGFR2 signaling could account for the residual cell survival and proliferation. And second, as VEGF can promote this cellular effect through FLT1 and studies have demonstrated that a dose of 50nM SU5416 did not inhibit FLT1 signaling [26] it is possible that VEGF-FLT1 signaling could elicit some effect on endothelial cell survival and proliferation. Collectively, the results demonstrated that SB623-derived trophic factors support the proliferation and survival of nutrient deprived endothelial cells and implicated VEGF as a major player in this process.

To restore nutrient and oxygen supply to the brain after stroke, the vasculature must be reinstated, starting with endothelial cell migration and sprouting. Using the HUVEC vascular tube formation assay which measures cell migration and invasion, we demonstrated that vascular tube formation was enhanced by conditioned medium from both SB623 cells and parental MSCs when compared to negative control. We next investigated the angiogenic activity of SB623-derived CM in a rat 3D aortic ring assay; this angiogenic model is more physiologically relevant as it contains both endothelial and vascular smooth muscle cells. One of the complications of the aortic ring assay is that there are endogenous angiogenic factors released by cells after preparation of the aortic rings [27]. To minimize the levels of endogenous factors, we therefore pre-cultured the aortic rings in serum-free endothelium medium for one week followed by a 24-hour culture in α-MEM, a medium containing ascorbic acid prior to use in angiogenic assay. Addition of SB623 cell- and MSC-CM both enhanced endothelial cell sprouting from the aortic rings. Of note, there was a higher degree of branching per sprout in conditions treated with SB623 cell-CM compared to MSC-CM. Branching of endothelial sprouts is important in establishing a complex vascular system; increased branching would provide a wider area of oxygen and nutrient supply to the brain. To determine the role of VEGF-KDR signaling role in endothelial cell migration and sprouting, 50nM SU5416 was added to both the HUVEC vascular tube formation assay and the aorting ring assay. In both models, this low dose of SU5416 completely reversed the migrating- and sprout-inducing activity of SB623 cell-CM. This is consistent with an earlier study showing that VEGF is a key regulator of endothelial cell migration and sprouting during angiogenesis and that this process is most efficient through KDR, not FLT1 [26].

It is interesting that SB623 cell-CM was superior to MSC-CM in promoting angiogenesis in the rat aortic ring assay, and not in the HUVEC assay. This observation is likely due to differences in the two assays. For instance, in the HUVEC assay, the only cells responsive to the exogenous CM are the human endothelial cells. In contrast, in the aortic ring assay, the responding cells include not only the endothelial cells but also the vascular smooth muscle cells and the macrophages, both of which are key players in angiogenesis. For instance, angiopoietin-2 which is consistently higher in SB623-CM than parental MSC-CM have been shown to increase the inherent angiogenic properties of macrophages [28]. CCL-2, a chemokine upregulated in SB623 cell-CM [17] is mitogenic to macrophages and thus, could synergize with angiopoietin-2 in amplifying the proangiogenic activity of aortic ring associated macrophages. IL-6 and IL-8, also upregulated in SB623-CM [17] have been shown to stimulate vascular smooth muscle cells [29,30], which in turn can amplify angiogenesis by secreting VEGF [31]. Future studies using co-cultures of HUVECs with smooth muscle cells and/or HUVECs with macrophages may give better insights into the mechanism of actions of SB623 cell-CM in the rat aortic ring assay.

## Conclusions

A panel of angiogenic paracrine factors differentially secreted by SB623 cells and the parental MSCs was identified using the Quantikine Angiogenesis Array. In the HUVEC assay and the aortic ring assay, SB623-conditioned medium is equally as potent in promoting endothelial tube formation and endothelial cell sprouting as MSC-conditioned medium. This functional aspect of SB623 cells may be one of the mechanisms by which SB623 cell transplantation promotes repair and regeneration in experimental stroke.

## Abbreviations

FLT-1: fms-related tyrosine kinase 1 or vascular endothelial growth factor receptor-1; HUVECS: human umbilical vein endothelial cells; MSCs: marrow stromal cells; MSC-CM: marrow stromal cell-conditioned medium; NICD: notch intracellular domain; RGF-BME: reduced growth factor-basement membrane extract; SB623: NICD-transfected marrow stromal cells; SB623-CM: NICD-transfected marrow stromal cell-conditioned medium; SU5416: VEGFR kinase inhibitor; VEGF: vascular endothelial growth factor; VEGFR: vascular endothelial growth factor receptor; VEGFR2: vascular endothelial growth factor receptor-2.

## Competing interests

All authors are employees of SanBio Inc.

## Authors’ contributions

MD: performed HUVECs and aortic ring assays, wrote the manuscript. CCT: performed Quantibody Angiogenic array and quantified angiogenic results, wrote the manuscript. MM: directed the production of SB623 cells and MSCs, edited the manuscript. CCC: supervised the research, edited the manuscript. All authors read and approved the final manuscript.

## Authors’ information

MD: Scientist at SanBio Inc.

CCT: Scientist at SanBio Inc.

MM: Senior Vice President of Production Development at SanBio Inc.

CCC: Executive Vice President of Research at SanBio Inc.
